# Tongxinluo Attenuates Myocardiac Fibrosis after Acute Myocardial Infarction in Rats via Inhibition of Endothelial-to-Mesenchymal Transition

**DOI:** 10.1155/2019/6595437

**Published:** 2019-06-16

**Authors:** Yujie Yin, Qian Zhang, Qifei Zhao, Guoyuan Ding, Cong Wei, Liping Chang, Hongrong Li, Hongying Bei, Hongtao Wang, Junqing Liang, Zhenhua Jia

**Affiliations:** ^1^Graduate School, Hebei University of Chinese Medicine, Shijiazhuang 050090, Hebei, China; ^2^Graduate School, Hebei Medical University, Shijiazhuang 050017, Hebei, China; ^3^Key Laboratory of State Administration of Traditional Chinese Medicine (Cardio-Cerebral Vessel Collateral Disease), Shijiazhuang 050035, Hebei, China; ^4^Hebei Yiling Pharmaceutical Research Institute, Shijiazhuang 050035, China; ^5^State Key Laboratory of Collateral Disease Research and Innovation Medicine, Shijiazhuang 050035, China; ^6^Department of Cardiology, Affiliated Yiling Hospital of Hebei University of Chinese Medicine, Shijiazhuang 050091, Hebei, China

## Abstract

Endothelial-to-mesenchymal transition (EndMT) is an essential mechanism in myocardial fibrosis (MF). Tongxinluo (TXL) has been confirmed to protect the endothelium against reperfusion injury after acute myocardial infarction (AMI). However, whether TXL can inhibit MF after AMI via inhibiting EndMT remained unknown. This study aims to identify the role of EndMT in MF after AMI as well as the protective effects and underlying mechanisms of TXL on MF. The AMI model was established in rats by ligating left anterior descending coronary artery. Then, rats were administered with high- (0.8 g·kg^−1^·d^−1^), mid- (0.4 g·kg^−1^·d^−1^), and low- (0.2 g·kg^−1^·d^−1^) dose Tongxinluo and benazepril for 4 weeks, respectively. Cardiac function, infarct size, MF, and related indicators of EndMT were measured. In vitro, human cardiac microvascular endothelial cells (HCMECs) were pretreated with TXL for 4 h and then incubated in hypoxia conditions for 3 days to induce EndMT. Under this hypoxic condition, neuregulin-1 (NRG-1) siRNA were further applied to silence NRG-1 expression. Immunofluorescence microscopy was used to assess expression of endothelial marker of vWF and fibrotic marker of Vimentin. Related factors of EndMT were determined by Western blot analysis. TXL treatment significantly improved cardiac function, ameliorated MF, reduced collagen of fibrosis area (types I and III collagen) and limited excessive extracellular matrix deposition (mmp2 and mmp9). In addition, TXL inhibited EndMT in cardiac tissue and hypoxia-induced HCMECs. In hypoxia-induced HCMECs, TXL increased the expression of endothelial markers, whereas decreasing the expression of fibrotic markers, partially through enhanced expressions of NRG-1, phosphorylation of ErbB2, ErbB4, AKT, and downregulated expressions of hypoxia inducible factor-1a and transcription factor snail. After NRG-1 knockdown, the protective effect of TXL on HCMEC was partially abolished. In conclusion, TXL attenuates MF after AMI by inhibiting EndMT and through activating the NRG-1/ErbB- PI3K/AKT signalling cascade.

## 1. Introduction

The cardiac remodeling and heart failure induced by acute myocardial infarction (AMI), as the dominating pathogenesis of chronic heart failure, has emerged as an increasingly serious health problem [1, 2]. Myocardial fibrosis (MF), featured by myocardial fibroblast accumulation, and deposition of extracellular matrix, is associated with sparseness of cardiac microvessels [3]. It is not only the main pathological marker of cardiac remodeling, but also the contributor to cardiac dysfunction [4, 5]. Despite available drug treatment strategy for MF, there is still an urgent need for development of new therapies to prevent and reverse MF after AMI.

Studies have shown that cardiac myofibroblasts are derived from resident fibroblasts, bone marrow-derived fibroblasts, and epithelial cells undergoing epithelial-to-mesenchymal transition (EMT) [6, 7]. Endothelial-to-mesenchymal transition (EndMT) is one subgroup of EMT, which is characterized by the gradual loss of endothelial phenotype and simultaneous acquirement of mesenchymal or fibroblastic features by endothelial cells [8]. As an activated form of myofibroblasts, fibroblasts are capable of synthesizing extracellular matrix. Under the pathological conditions, EndMT is involved in fibrosis formation in various fibrotic diseases such as myocardial fibrosis [7], organ fibrosis [9], solid tumors [10], pulmonary hypertension [11], atherosclerosis [12], and cerebral cavernous vascular malformations [13]. It is reported that hypoxia plays an important role in the process of EndMT [14]. However, little is known about the mechanisms for hypoxia-induced EndMT.

TXL, a traditional Chinese medicine available in capsule form, has been widely used for treatment of cardiovascular diseases. It is made from 12 distinct herbs including* Radix ginseng, Hirudo, Radix paeoniae rubra, Periostracum cicadae, scorpion, Eupolyphaga Steleophaga, Scolopendra, Lignum santali albi, Lignum dalbergiae odoriferae, frankincense, Zizyphus jujube benevolence, and Borneolum syntheticum* registered in the State Food and Drug Administration (SFDA) of China in 1996 [15]. Previous studies have revealed that TXL can stabilize atherosclerotic plaques, lower lipid levels, improve endothelial function, promote vasodilation, inhibit inflammation and apoptosis [16], and enhance angiogenesis [17]. Furthermore, TXL has also been demonstrated to ameliorate acute coronary syndrome and unstable angina pectoris and reduce myocardial no-reflow and reperfusion injury by preserving endothelial function [18]. However, the role of TXL in rat MF after AMI is rarely studied.

Neuregulins (NRGs), a member of the epidermal growth factor family, consist of neuregulin 1 (NRG-1), NRG-2, NRG-3, and NRG-4, which share a common epidermal growth factor-like domain. NRG-1 is expressed in the endocardium and cardiac microvascular endothelium [19]. Receptors for the cardiac-specific NRGs are the ErbB family of tyrosine kinase transmembrane receptors composed of ErbB2, ErbB3, and ErbB4. ErbB4 binds to its ligand NRG-1 and forms a heterodimer with ErbB2, which further activates the intracellular kinase pathway. Autophosphorylation of tyrosine residues could recruit effective molecules [20] and direct downstream signalling pathways to participate in myocardial protection in a paracrine and autocrine fashion [21–23]. NRG-1/ErbB signaling has been demonstrated to involve in cardioprotective effects in heart, including attenuating cardiac dysfunction [24], promoting neovascularization in the ischemic myocardium [25], inhibiting apoptosis [19], and regulating endoplasmic reticulum stress [25]. Despite the advances in cardioprotective effects of NRG-1, the function of NRG-1 in EndMT is poorly understood.

This study was designed to investigate the role and potential mechanisms of EndMT in MF after AMI. Furthermore, whether TXL could ameliorate MF and its effects on EndMT were also investigated in an AMI rat model and in hypoxia-treated HCMECs.

## 2. Materials and Methods

### 2.1. Ethics Statement

Male Sprague-Dawley (SD) rats (200–220g; approximately 6 weeks old) were used in this study. All animals were kept at the Key Laboratory of State Administration of Traditional Chinese Medicine (Cardio-Cerebral Vessel Collateral Disease) (Shijiazhuang, China). All procedures with animals were conducted under the guidelines on the Care and Use of Laboratory Animals for biomedical research published by National Institutes of Health (No. 85-23, revised 1996) and according to the ethical guidelines of the Ethics Committee of Hebei Yiling Pharmaceutical Research Institute (No. N2018026).

### 2.2. Preparation of TXL

TXL ultrafine powder was provided by Shijiazhuang Yiling Pharmaceutical Corporation (Shijiazhuang, China). After dissolving TXL powder in serum-free Endothelial Cell Medium (ECM), the mixture was sonicated and centrifuged at 10000×g for 10 min. After centrifugation, the precipitate was dried at 60°C and the supernatant was filtered (0.22 *μ*m pore size) to calculate an accurate weight of the dissolved ingredients [15].

### 2.3. Animals and Treatments

SD rats were randomly divided into 6 groups (n=10 per group) and were treated by intragastric administration as follows for 4 weeks: (i) sham operation; (ii) model group; (iii) low-dose TXL group; (iv) middle-dose TXL group; (v) high-dose TXL group; (vi) benazepril group. On day 1, SD rats were intraperitoneally anesthetized by 10% chloral hydrate (350mg·kg^−1^ body weight) [26], and then tracheal intubation was performed. The left anterior descending coronary (LAD) was occluded permanently to induce experimental myocardial fibrosis, while sham group was established by the same procedure but without LAD ligation. From days 1 to 28, rats were given a gavage of low-dose TXL group (0.2 g·kg^−1^·d^−1^), middle-dose TXL group (0.4 g·kg^−1^·d^−1^), high-dose TXL group (0.8 g·kg^−1^·d^−1^), and benazepril (10mg·kg^−1^·d^−1^) once a day. After intragastric administration, SD rats were intraperitoneally anesthetized by 10% chloral hydrate (350mg·kg^−1^ body weight) for 5-10 minutes. No SD rat exhibited signs of peritonitis after anaesthesia. When the breath of the rats was even and steady, the rats' four toes were gently clamped with tweezers to observe pain reflex. If there was no pain reflex, the rats were determined to be in a state of deep anaesthesia. Cardiac function was detected by echocardiography. Then, blood sample (6-8 mL each rat) was taken from the abdominal aorta while they were still alive for a scientific purpose. After that, the method of cervical dislocation causing instantaneous unconsciousness was used for the euthanasia of SD rats [27]. Death verification was confirmed by cessation of heartbeat and respiration and absence of reflexes. After the rats stopped breathing, the heart sample was collected.

### 2.4. Echocardiographic Measurements

Echocardiography was performed in rats using a 15 MHz ultrahigh frequency scan head (MyLab Five, Esaote, Italy). Specifically, M-mode tracings of the left ventricles (LVs) were recorded at the parasternal long-axis view including left ventricular end-diastolic dimension (LVEDd) and left ventricular end-systolic dimension (LVESd), which were used to calculate left ventricular ejection fraction (EF) and left ventricular fractional shortening (FS). Measurement and analysis were performed by the same experienced investigator in a blinded fashion.

### 2.5. Masson Trichrome Staining

Rat myocardial tissues were fixed in 4% paraformaldehyde, routinely paraffin-embedded, and cut into 4 *μ*m thick sections. Tissue sections were stained with Masson's trichrome and examined using light microscopy. The collagen fiber was stained blue. Different fields were randomly selected to measure the fibrotic area under the microscope by Image Pro Plus (Media Cybernetics, Inc., Bethesda, MD, USA). The fibrotic area was calculated as the ratio of the area of fibrosis (stained in red) to the total area of fibrosis and normal cardiomyocytes area (stained in red).

### 2.6. Cell Culture and Treatment

Human cardiac microvascular endothelial cells (HCMECs) (ScienCell Research Laboratories, California, USA) were maintained in complete ECM, which included 5% fetal bovine serum (FBS), 1% endothelial cell growth supplement (ECGS), and 1% penicillin/ streptomycin (P/S), and incubated in a 37°C, 5% CO2 incubator according to the manufacturer's instructions. When the confluency reached 90%, cells were washed with DPBS, digested with 0.25% trypsin-EDTA, and subcultured at a ratio of 1:3 in a Petri dish precoated with 4mL Bovine Plasma Fibronectin (BPF) (ScienCell Research Laboratories).

HCMECs were cultured in normoxic conditions to 30-35% confluency and then divided into 5 groups: (i) control group: HCMECs were cultured under normoxic conditions; (ii) hypoxia group: HCMECs were cultured under hypoxia conditions (1% O2, 5% CO2, and 94% N2) (Thermo Fisher Scientific) for 3 days to establish hypoxia-induced EndMT [28]; (iii) hypoxia + high-dose TXL group: HCMECs were pretreated with TXL (600 ug·ml^−1^) for 4 h and cultured under hypoxia conditions for 3 days; (iv) hypoxia + middle-dose TXL group: HCMECs were pretreated with TXL (400 ug·ml^−1^) for 4 h and cultured under hypoxia conditions for 3 days; (v) hypoxia + low-dose TXL group: HCMECs were pretreated with TXL(200 ug·ml^−1^) for 4 h and cultured under hypoxia conditions for 3 days.

### 2.7. Cell Transfection by Small-Interfering RNA

The siRNAs targeting NRG-1 (siRNA-1380, 1626, 1987) were synthesized by Shanghai GenePharma Cooperation. The NRG-1 siRNA sequences were shown in Table 1. After HCMECs were 70–75% confluent, they were transfected with mixture of diluted siRNAs targeting NRG-1 (with red fluorescence protein labelled) and diluted Lipofectamine ™ 3000. For control group, only Lipofectamine ™ 3000 diluted in Opti-MEM medium was used [19]. Flow cytometry and Western blot were used to evaluate the success rate of transfection and inhibition after transfected with three siRNAs NRG-1 in HCMECs for 24 hrs, respectively. According to the results of flow cytometry and Western blot, siRNA-1626 had the optimal inhibitory effect on NRG-1 expression compared to negative-transfected conditions (data not shown). Thus, siRNA-1626 was used in the following experiments. The transfected HCMECs were placed into hypoxia incubator for 3 days after they were incubated with or without TXL for 4h. Specifically, HCMECs were divided into 5 groups: (i) control group; (ii) hypoxia group; (iii) hypoxia + NRG-1 siRNA group; (iv) hypoxia + TXL group; (v) hypoxia + NRG-1 siRNA + TXL group. The control group was cultured in normoxic conditions.

### 2.8. Immunofluorescence Staining

After pretreated HCMECs reached 70% confluence, they were washed with DPBS, fixed in 4% paraformaldehyde for 15 min, and permeabilized with solution containing 0.1% Triton X-100 for 5 min. After being blocked with goat serum for 1h at room temperature, cells were further incubated with primary antibodies (anti-vWF, (Alexa Fluor® 488) ab195028, 1:100; anti-Vimentin, ab92547, 1:400), respectively, at 4°C overnight. After washing, cells were incubated with secondary antibodies (Goat Anti-Rabbit, ab96899, 1:200). Cell nuclei were stained using DAPI for another 15 min and visualized using a ZEISS confocal microscopy (Oberkochen, Germany) [29, 30].

### 2.9. Real-Time Polymerase Chain Reactions (PCR)

Total RNA was extracted from rat myocardial tissue by TRIzol reagent and measured by NANODROP 2000 (Thermo Scientific) [31]. Subsequently, reverse-transcription was performed with Prime-Script™ RT Reagent Kit according to manufacturer's instructions. Quantitative PCR was carried out using 7900 Real-Time PCR System (Applied Biosystems, USA). *β*-actin was used as an internal reference. Primer sequences used for the RT-PCR are listed in Table 2.

### 2.10. Western Blotting Analysis

Protein concentration of HCMECs and rat myocardial tissue was determined with BCA protein assay kit before electrophoresis on SDS-PAGE. Proteins were then transferred onto Nitrocellulose Blotting membrane (Life Sciences, Mexico). After blocking with Odyssey® blocking Buffer (LI-COR, Lincoln USA) for 2h at 37°C, membranes were incubated with primary antibodies (Collagen I, Collagen III, MMP-2, MMP-9, CD31,*α*-SMA, VE-cadherin, FSP-1, Vimentin, HIF-1*α*, NRG-1, phospho-ErbB2, phospho-ErbB4, p-AKT, snail, *β*-actin) (Table 3) at 4°C overnight. After being rinsed with TBST 3 times, the membranes were further incubated with secondary antibodies at 37°C for 1 hr. Finally, the membranes were being rinsed with TBST 3 times before scanned by Odyssey (LI-COR, Lincoln USA). The *β*-actin was used as a corresponding internal control.

### 2.11. Statistical Analysis

Data are expressed as mean±SD. Comparisons among multiple groups were conducted using one-way analysis of variance (ANOVA) and considered statistically significant when* P*-values were less than 0.05. All analyses were performed using SPSS 19.0 or GraphPad Prism 5.0.

## 3. Results

### 3.1. TXL Improved Cardiac Function in AMI Rats

**Figure 1:**
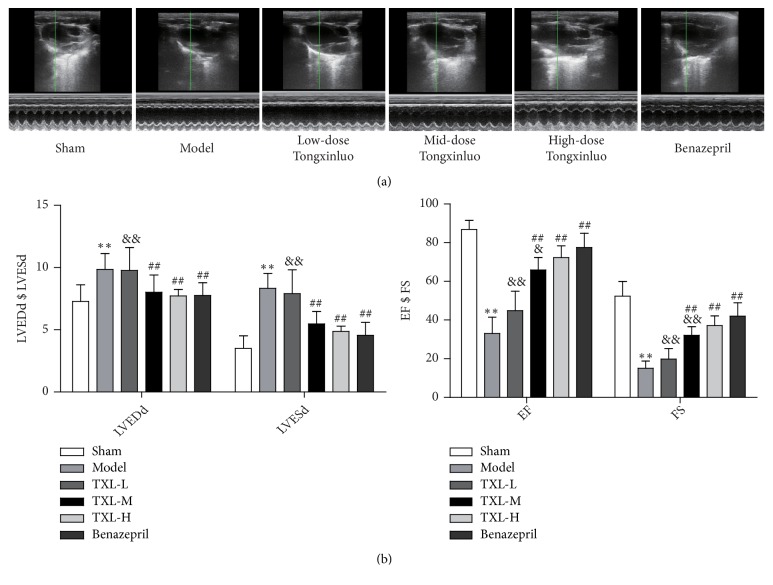
*Echocardiographic data show that TXL improves cardiac function of rat after AMI*. (a) Cardiac function 4 weeks after AMI was examined by echocardiography. M-mode tracings of the left ventricles were recorded at the parasternal long-axis view. (b) The M-mode pictures were analyzed. LVEDD and LVESD were increased whereas EF and FS were decreased in model group. TXL improves cardiac function including reduced LVEDD, LVESD and preserved left ventricular EF and FS compared with model group. n=10, *∗∗*P<0.01 vs. control group; ^##^ P<0.01 vs. model group;  ^&^ P <0.05  ^&&^ P <0.01 vs. benazepril group.

Rats were subjected to LAD ligation and hereafter treated with TXL at low, middle, and high dose and benazepril for 4 weeks, respectively. Echocardiography was used to examine cardiac function 4 weeks after AMI (Figure 1(a)). The low-dose TXL had no significant effect on cardiac function; however, mid-dose and high-dose TXL significantly decreased LVEDD, LVESD and increased EF, FS as compared with model group. Benazepril also improved cardiac function (Figures 1(a) and 1(b)).

### 3.2. TXL Attenuated MF after AMI in Rats

**Figure 2:**
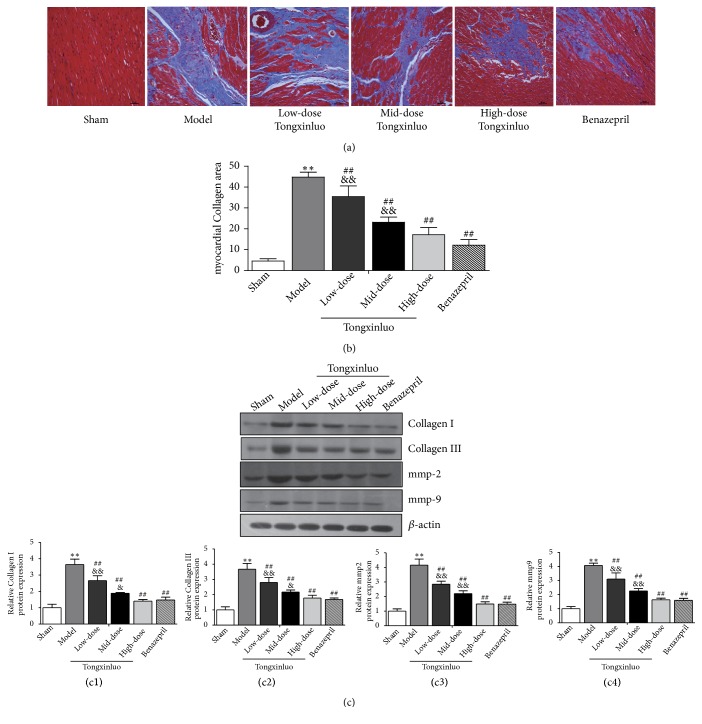
*Effects of TXL on MF of rat after AMI*. (a) Myocardial fibrosis was detected by Masson trichrome staining. Image Pro Plus was performed to measure fibrotic fraction, which was calculated as the ratio of the area of fibrosis (stained in red) to the total area of fibrosis and normal cardiomyocytes area (stained in red). Scale bar: 50*μ*m. (b) Effects of TXL on myocardial collagen areas.  ^*∗∗*^P<0.01 vs. control group; ^##^P<0.01 vs. Model group; ^&&^P <0.01 vs. benazepril group. (c) Effects of TXL on the protein expressions of MMP-2, MMP-9, types I and III collagen in the MF were determined by Western blotting analysis. (c1)–(c4) showed corresponding column chart. n=3,  ^*∗∗*^P<0.01 vs. control group; ^##^P<0.01 vs. Model group; ^&^P <0.05 ^&&^P <0.01 vs. benazepril group.

As well know, MF is a definite feature of cardiac remodeling after AMI. Masson trichrome staining was performed to analyze interstitial fibrosis. As shown in Figure 2(a), there was a large area of blue-stained collagen fiber between myocardial cells. The degree of MF was significantly declined in rats receiving TXL treatment compared with model group (Figure 2(b)). Furthermore, protein expression of MMP-2 and MMP-9, which are contributors to cardiac remodeling [26] after AMI, showed a distinct decrease in TXL and benazepril-treated rats. Expression of types I and III collagens, which are well recognized as fibrosis-related factors, were downregulated in TXL and benazepril-treated rats compared with model group (Figure 2(c)). Moreover, there was no significant difference between the high dose of TXL and benazepril group. Collectively, the results indicate that TXL attenuated MF after AMI in a dose-dependent manner.

### 3.3. Effects of TXL on EndMT during MF in Rats

**Figure 3:**
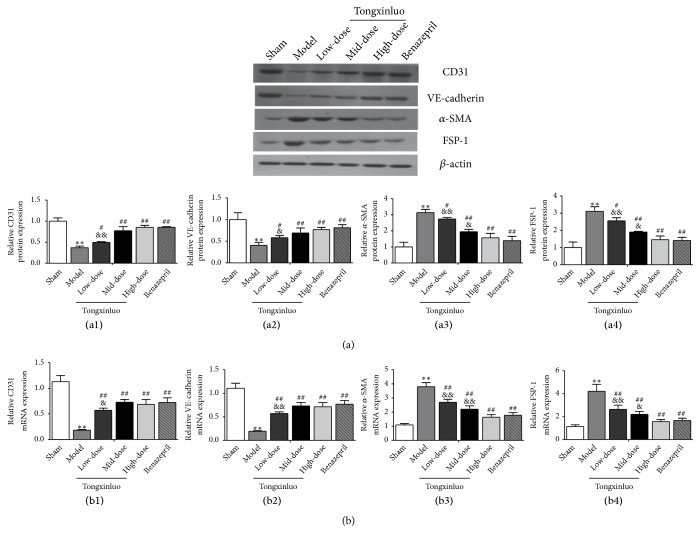
*Effect of TXL on EndMT of the MF in vivo*. (a) Effects of TXL on protein expression of endothelial markers (CD31 and VE-cadherin) and mesenchymal markers (*α*-SMA and FSP-1) in the MF were determined by Western blotting analysis. (a1)–(a4) showed corresponding column chart. n=3,  ^*∗∗*^P<0.01 vs. control group; ^#^P<0.05 ^##^P<0.01 vs. Model group; ^&^P <0.05 ^&&^P <0.01 vs. benazepril group. (b) Effects of TXL on expression of CD31, VE-cadherin, *α*-SMA, and FSP-1 at mRNA levels were analyzed by qRT-PCR. (b1)–(b4) showed corresponding column chart. n=4,  ^*∗*^P<0.05 or  ^*∗∗*^P<0.01 vs. control group; ^##^P<0.01 vs. Model group; ^&^P <0.05 ^&&^P <0.01 vs. benazepril group.

EndMT plays a crucial role in endothelial injury [32]. To further evaluate the influence of EndMT on MF, we detected the content of specific endothelial markers such as CD31 and VE-cadherin and mesenchymal markers such as *α*-SMA and FSP-1 by Western blot and qRT-PCR. As shown in Figures 3(a) and 3(b), in model group, the level of CD31 and VE-cadherin was progressively decreased whereas that of *α*-SMA and FSP-1 was increased at both protein and mRNA levels, which indicates that EndMT participates in MF. However, protein level of CD31 and VE-cadherin showed a distinct increase whereas *α*-SMA and FSP-1 levels were significantly decreased after TXL treatment. The effect was not significantly different between high-dose TXL and benazepril group (Figures 3(a) and 3(b)). Therefore, TXL relieves EndMT during MF.

### 3.4. TXL Protects HCMECs against EndMT Induced by Hypoxia

**Figure 4:**
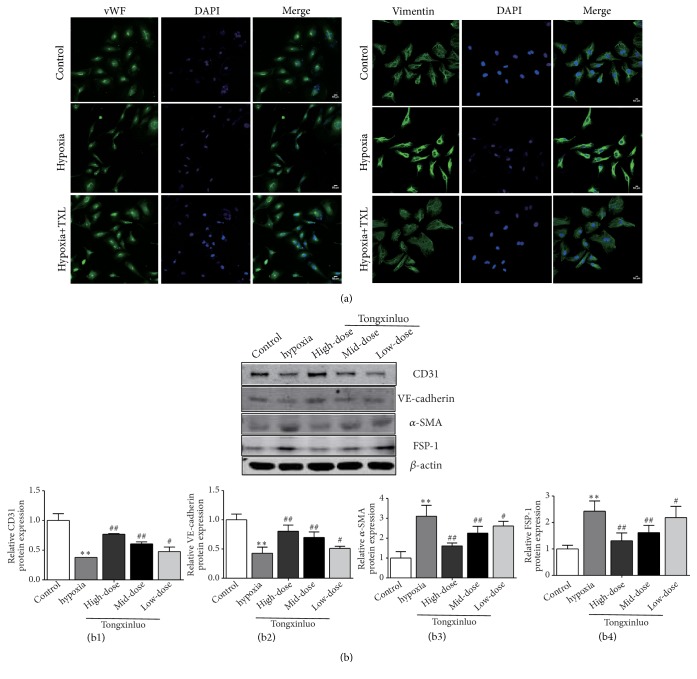
*TXL protects HCMECs against EndMT induced by hypoxia*. (a) Immunofluorescence staining showed the endothelial markers vWF and mesenchymal markers Vimentin in the control group, treated with hypoxia (1% O2) for 3 days and cotreated with TXL (600 ug·ml^−1^) and hypoxia (1% O2) for 3 days. Nuclear staining with DAPI (blue), scale bar: 50*μ*m. (b) Effects of TXL on protein expression of CD31, VE-cadherin, *α*-SMA, and FSP-1 in vitro were determined by Western blotting analysis. (b1)–(b4) showed corresponding column chart. n=3, *∗∗*P<0.01 vs. control group; ^#^P<0.05 ^##^ P<0.01 vs. hypoxia group.

The HCMECs were incubated in hypoxia conditions (1% O2) for 3 days, to establish a model of EndMT in vitro. Immunofluorescence staining showed that HCMECs displayed a typical cobblestone morphology and expressed endothelial marker gene vWF under normoxic conditions. When exposed to hypoxia conditions, HCMECs lost their cobblestone feature, presented a dispersed, spindle-shaped appearance, and expressed mesenchymal marker gene Vimentin (Figure 4(a)). Similarly, the relative protein expression levels of CD31 and VE-cadherin, as determined by Western blot, were downregulated, while the protein expression levels of *α*-SMA and FSP-1 were upregulated in the hypoxia group (Figure 4(b)), which indicate that hypoxia could induce the occurrence of EndMT in HCMECs.

Furthermore, we observed changes in EndMT-related factors in HCMECs treated with TXL. Immunofluorescence staining revealed that the hypoxia-induced upregulation of Vimentin was inhibited by TXL (Figure 4(a)). HCMECs treated with TXL presented cobblestone appearance and had upregulation of vWF. We monitored the changes of expression of endothelial and mesenchymal marker genes and the results showed that the hypoxia-induced upregulation of *α*-SMA and FSP-1 and downregulation of CD31 and VE-cadherin were reversed by TXL (Figure 4(b)). TXL could inhibit EndMT induced by hypoxia, which is consistent with the results of TXL inhibiting EndMT in the process of MF in vivo.

### 3.5. TXL Activates the NRG-1/ErbB-PI3K/AKT Signaling Pathway

**Figure 5:**
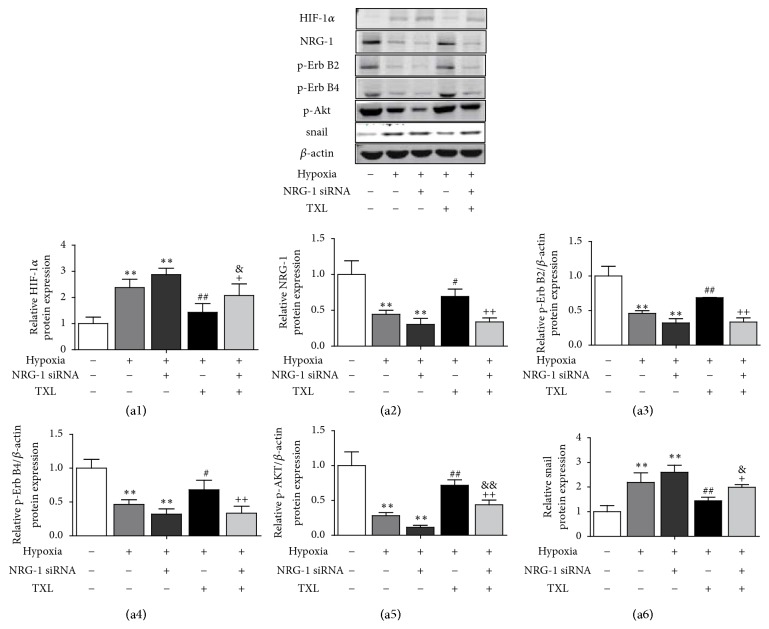
*NRG-1/ErbB-PI3K/AKT signaling pathway was activated by TXL*. Effects of TXL on protein expression of NRG-1, p-ErbB2, p-ErbB4, p-AKT, and snail in vitro were determined by Western blotting analysis. (a1)–(a6) showed corresponding column chart. *β*-actin expressions served as internal control. n=3, *∗∗*P<0.01 vs. control group;^ #^ P<0.05 ^##^ P<0.01 vs. hypoxia group;  ^&^ P<0.05 ^&&^ P<0.01 vs. hypoxia +NRG-1 siRNA group; ^+^ P<0.05 ^++^ P<0.01 vs. hypoxia +TXL group.

We subsequently knocked down the expression of NRG-1 in HCMECs using small-interfering RNA. Western blot was used to detect protein levels (Figure 5). In the absence of NRG-1 siRNA, the levels of NRG-1, p-ErbB2, p-ErbB4, and p-AKT were decreased and the level of snail, a zinc-finger binding transcription factor of EndMT, was increased in hypoxia treatment group. After NRG-1 siRNA treatment, we found the protein level of NRG-1, p-ErbB2, p-ErbB4 and p-AKT significantly decreased, and snail level was further increased than control group. However, TXL pronouncedly elevated the levels of NRG-1, p-ErbB2, p-ErbB4 and p-AKT and reduced the level of snail in HCMECs under hypoxia. Nevertheless, the activating effect of TXL on NRG-1/ErbB-PI3K/AKT signaling cascades was reversed by NRG-1 siRNA. Moreover, NRG-1 siRNA also partially prevented the inhibitory effect of TXL on snail.

### 3.6. Knockdown of NRG-1 Enhances Hypoxia-Induced EndMT of HCMECs

**Figure 6:**
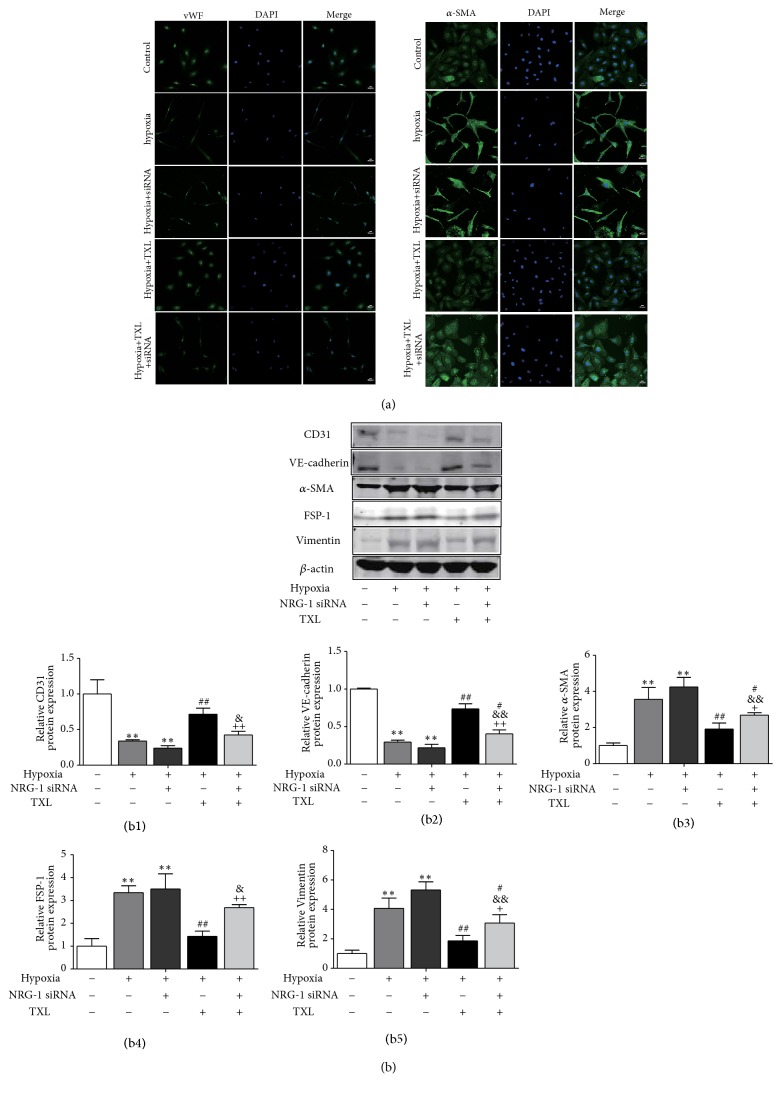
*TXL regulates EndMT through NRG-1/ ErbB signaling pathway in the presence of hypoxia*. (a) Immunofluorescence for EndMT markers vWF and *α*-SMA in HCMECs treated with TXL and NRG-1 siRNA in the presence of hypoxia. Nuclear staining with DAPI (blue fluorescence). Scale bar: 50*μ*m. (a) Effects of TXL on endothelial markers CD31 and VE-cadherin and mesenchymal markers *α*-SMA, FSP-1 and Vimentin with or without pretreatment with NRG-1 siRNA in the presence of hypoxia (1% O2) for 3 days. (b1)–(b5) showed corresponding column chart. n=3, *∗∗*P<0.01 vs. control group; ^#^ P<0.05 ^##^ P<0.01 vs. hypoxia group;  ^&^ P<0.05 ^&&^ P<0.01 vs. hypoxia +NRG-1 siRNA group; ^+^ P<0.05 ^++^ P<0.01 vs. hypoxia +TXL group.

We further explored the role of NRG-1 in hypoxia-induced EndMT. As anticipated, we found that NRG-1 siRNA prompted the morphology changes of HCMECs from typical cobblestone morphology to spindle morphology, and induced downregulation of vWF and upregulation of *α*-SMA (Figure 6(a)). Additionally, NRG-1 siRNA reduced the protein level of CD31 and VE-cadherin, and upregulated the protein level of *α*-SMA, FSP-1 and Vimentin (Figure 6(b)). The data demonstrated that knockdown of NRG-1 enhances EndMT of HCMECs.

### 3.7. NRG-1/ErbB Pathway Is Involved in TXL-Mediated Inhibition of EndMT

We further investigated whether the NRG-1/ErbB- PI3K/AKT signaling cascades indeed mediate the inhibition effect of TXL on EndMT. Immunofluorescence staining results clearly demonstrated that pretreatment with NRG-1 siRNA suppressed the upregulation of vWF and the downregulation of *α*-SMA at protein level in the presence of TXL treatment (Figure 6(a)). Similar results were obtained at protein levels that after NRG-1 siRNA treatment, TXL lost its ability to upregulate the protein level of endothelial marker genes (CD31 and VE-cadherin) and downregulate that of mesenchymal marker genes (*α*-SMA, FSP-1, and Vimentin) (Figure 6(b)). Our results prompt that NRG-1/ErbB- PI3K/AKT signaling cascades participate in the protective effect of TXL on HCMEC.

## 4. Discussion

This study used an in vivo and in vitro model, respectively, to elucidate the effects and potential mechanism of TXL on infarcted hearts. Several observations were particularly noteworthy, as summarized below: (i) TXL could improve cardiac function and attenuate the degree of ischemic-associated MF of rats after AMI via EndMT. (ii) TXL suppressed the hypoxia-induced EndMT in HCMECs. (iii) NRG-1/ErbB-PI3K/AKT signaling pathway was participated in the hypoxia-induced EndMT as a negative feedback mechanism. (iv) TXL prevented HCMECs from EndMT through NRG-1/ErbB-PI3K/Akt signaling pathway activation under hypoxia.

Cardiac microvascular endothelial damage may result in reduced microvascular perfusion, cardiac remodeling, and impaired chronic heart function [33, 34]. MF is the main pathological marker of cardiac remodeling. This research used permanent LAD ligation to establish MF model. Cardiac function, infarct size, and MF were measured by echocardiography and Masson trichrome staining. Indeed, we observed impaired cardiac function, increased fibrillar collagens (types I and III collagens), and excessive extracellular matrix deposition (mmp-2 and mmp-9) in model establishment. However, these alterations were reversed by TXL, confirming it could improve cardiac function and attenuate the degree of ischemic-associated MF. The antifibrosis effects of TXL on AMI rat may be related to its protective effect on cardiac microvascular endothelial damage during ischemic heart disease.

TXL, a traditional Chinese medicine, has been used for treating unstable angina pectoris and acute coronary syndrome in China [35]. It is indicated that TXL could protect the endothelial function and has anti-inflammation and antiapoptosis effects [16] and enhance angiogenesis. To better understand the effects of TXL on antifibrosis, we further explored the potential mechanisms of TXL from the perspective of protecting cardiac microvascular endothelium.

EndMT means that cardiac/vascular endothelial cells undergo mesenchymal transition. Excessive deposition of extracellular cell matrix mediated by integrated fibroblasts is important in promoting MF. Study has reported that in pressure-overloaded mice model, 27%–35% of fibroblasts are derived from endothelial cells (ECs) undergoing EndMT [7]. Thus, the effect of EndMT on myocardial fibrosis is increasingly important. In the present study, in vivo experiments demonstrated that AMI could induce EndMT in rats as showed by the increases in mesenchymal cell marker genes and marked decreases in the expressions of endothelial cell marker genes at both protein and mRNA levels, revealing that EndMT may play a crucial role in ischemia-induced MF. However, rats treated with TXL could enhance cardiac microvascular endothelial properties and decrease expression of mesenchymal cell markers. The EndMT-related alterations in MF model were reversed by TXL, suggesting that TXL prevents ischemic-associated MF after AMI via inhibiting the EndMT.

Hypoxia is involved in the pathogenesis of cardiovascular diseases. Researches have shown that hypoxia could induce occurrence of EndMT in various diseases [14, 36]. Injured ECs migrate to the perivascular or cardiac stroma via EndMT and transform into myofibroblasts under hypoxia conditions [37]. Here, we established a hypoxia-induced EndMT model in HCMECs in vitro and demonstrated that hypoxia could induce EndMT in HCMECs as indicated by acquiring an elongated shape and an end-to-end polarity while losing the cobblestone appearance of endothelial cells. Furthermore, we explored the influences of TXL on hypoxia-induced EndMT in HCMECs. TXL inhibited EndMT. HCMECs treated with TXL presented cobblestone appearance and had upregulated expression of endothelial markers. In line with the in vivo results, TXL also could protect HCMECs against EndMT induced by hypoxia.

Research previously demonstrated that NRG-1/ErbB modulated endothelial function of cardiac microvascular endothelial cells in a PI3K/AKT-dependent manner [19]. Significantly impaired systolic function was observed in NRG-1 knockout mouse model of myocardial infarction, which was significantly improved after NRG-1 intervention [28]. Hadis Shakeri [38] et al. confirmed that NRG-1 played a protective role in vascular senescence. These findings also provide evidence that NRG-1 is essential for cardiovascular system diseases. Li et al. confirmed that under oxidative stress, the application of PI3K-specific inhibitors in HAECs induced EndMT; in contrast, this condition was reversed by the pretreatment of PI3K activators [39]. PI3K/AKT signaling pathway has been confirmed to play a crucial role in EndMT [40, 41]. However, the signaling cascades involved in hypoxia-induced EndMT remain unclear. In the present study, we found that the levels of NRG-1, p-ErbB2, p-ErbB4, and p-AKT were decreased in HCMECs under hypoxia. NRG-1 binds to ErbB receptors by autocrine signaling, induces ErbB2 and ErbB4 phosphorylation, and thereby activates the PI3K/AKT signaling pathway in HCMECs. Herein, the activation of AKT was abrogated by the application of NRG-1 siRNA in presence of hypoxia. PI3K/AKT signaling pathway is a downstream of NRG-1/ErbB signaling network, which contributes to cytoprotection of HCMECs. The level of snail, a zinc-finger binding transcription factor of EndMT, was increased in hypoxia group. The activation of snail could induce EndMT process in endothelial cells involved in MF [28]. TXL pronouncedly activated the NRG-1/ErbB- PI3K/AKT signaling cascades and increased the level of snail.

In order to clarify whether the NRG-1/ ErbB signaling indeed mediates the inhibition of EndMT by TXL, HCMECs were pretreated with NRG-1 siRNA to knockdown NRG-1 expression in the presence of hypoxia. As anticipated, we found that NRG-1 siRNA prompted the morphology changes of HCMECs into spindle morphology and induced downregulation of endothelial marker genes and upregulation of mesenchymal marker genes in presence of hypoxia, demonstrating that knockdown of NRG-1 enhances hypoxia-induced EndMT in HCMECs. Furthermore, we pretreated cells with NRG-1 siRNA and then with TXL. As anticipated, under such condition, the beneficial effects of TXL were reversed partially by application of NRG-1 siRNA, suggesting that TXL prevented HCMECs from EndMT through activating NRG-1/ErbB-PI3K/AKT signaling cascades under hypoxia.

The limitations of present study in terms of design must be acknowledged. For example, to test the NRG-1/ErbB-PI3K/AKT signaling cascades involved in hypoxia-induced EndMT, HCMECs should be pretreated with LY294002 to blockade of PI3K/AKT pathway. In addition, although specific gene knockdown was used in the present study, the relationship between EndMT and NRG-1/ErbB can be more accurately reflected by NRG-1 gene overexpression in HCMECs simultaneously. Moreover, the study of signaling pathways related to EndMT was only performed* in vitro*. Considering the complexity of environment* in vivo*, we should further extend research on the molecular mechanism of EndMT in MF* in vivo*. Further studies are warranted.

## 5. Conclusions

EndMT is an essential mechanism in MF after AMI. The present findings demonstrate that TXL attenuates cardiac remodeling and MF after AMI in rats and that TXL could produce cytoprotection against hypoxia by inhibiting the hypoxia-induced EndMT in HCMECs, activating the NRG-1/ErbB- PI3K/AKT signaling cascade, and limiting excessive extracellular matrix deposition.

## Figures and Tables

**Table 1:** Sequences for siRNAs against NRG-1.

siRNA	Sequences	
	Sense (5*ʼ*–3*ʼ*)	Antisense (5*ʼ*–3*ʼ*)
NRG-1 siRNA-1380	GGUCUGAACGAAACAAUAUTT	AUAUUGUUUCGUUCAGACCTT
NRG-1 siRNA-1626	CCGAAAGCCACUCUGUAAUTT	AUUACAGAGUGGCUUUCGGTT
NRG-1 siRNA-1987	GCUGCGGGAGAAGAAGUUUTT	AAACUUCUUCUCCCGCAGCTT

**Table 2:** RT-PCR primer sequences.

Genes	Forward (5'→3')	Reverse (5'→3')
CD31	CAGAAGGAAGAGACGGTGTTG	TTGACCACTTTGCCGATG
*α*-SMA	GATTATTGCTCCTCCAGAACG	CTTCGTCATACTCCTGTTTGCT
VE-cadherin	AATAAAGACCAGGTGACCACG	GATGGCGGGAACTTGTAATC
FSP-1	GGACAGCAACAGGGACAATG	CCCAACACTTCATCTGAGGAG
*β*-actin	GGTCATCACCATTGGCAA	GAGTTGAAGGTAGTTTCGTGGA

**Table 3:** Primary antibodies.

Primary antibodies	Companies	Dilution rate
Anti-Collagen I	Abcam, ab6308	1:1000
Anti-Collagen III	Abcam, ab7778	1:5000
Anti-MMP-2	Abcam, ab92536	1:1000
Anti-MMP-9	Abcam, ab76003	1:1000
Anti-CD31	Abcam, ab24590	1:1000
Anti-*α*-SMA	Abcam, ab32575	1:1000
Anti-VE-cadherin	Abcam, ab166715	1:1000
Anti-FSP-1	Abcam, ab197896	1:1000
Vimentin Rabbit mAb	Cell Signaling Technology, D21H3	1:1000
HIF-1*α* Rabbit mAb	Cell Signaling Technology, D2U3T	1:500
Anti-NRG-1	Abcam, ab180808	1:1000
Anti-phospho-ErbB2	Abcam, ab47262	1:500
Anti-phospho-ErbB4	Abcam, ab61059	1:500
Anti-p-Akt	Abcam, ab192623	1:1000
snail Rabbit mAb	Cell Signaling Technology, C15D3	1:1000
*β*-actin	Cell Signaling Technology	1:1000

## Data Availability

The datasets generated and analyzed during the current study are available from the corresponding author on reasonable request.

## References

[1] Heusch G., Libby P., Gersh B. (2014). Cardiovascular remodelling in coronary artery disease and heart failure. *The Lancet*.

[2] Vilahur G., Juan-Babot O., Peña E., Oñate B., Casaní L., Badimon L. (2011). Molecular and cellular mechanisms involved in cardiac remodeling after acute myocardial infarction. *Journal of Molecular and Cellular Cardiology*.

[3] van Putten S., Shafieyan Y., Hinz B. (2016). Mechanical control of cardiac myofibroblasts. *Journal of Molecular and Cellular Cardiology*.

[4] Cai J., Chen X., Chen X. (2017). Anti-Fibrosis effect of relaxin and spironolactone combined on isoprenaline-induced myocardial fibrosis in rats via inhibition of endothelial-mesenchymal transition. *Cellular Physiology and Biochemistry*.

[5] Travers J. G., Kamal F. A., Robbins J., Yutzey K. E., Blaxall B. C. (2016). Cardiac fibrosis: the fibroblast awakens. *Circulation Research*.

[6] Iwano M., Plieth D., Danoff T. M., Xue C., Okada H., Neilson E. G. (2002). Evidence that fibroblasts derive from epithelium during tissue fibrosis. *The Journal of Clinical Investigation*.

[7] Zeisberg E. M., Tarnavski O., Zeisberg M. (2007). Endothelial-to-mesenchymal transition contributes to cardiac fibrosis. *Nature Medicine*.

[8] Xiang Y., Zhang Y., Tang Y., Li Q. (2017). MALAT1 modulates TGF-beta1-induced endothelial-to-mesenchymal transition through downregulation of miR-145. *Cellular Physiology and Biochemistry*.

[9] Choi S.-H., Hong Z.-Y., Nam J.-K. (2015). A hypoxia-induced vascular endothelial-to-mesenchymal transition in development of radiation-induced pulmonary fibrosis. *Clinical Cancer Research*.

[10] Xiao L., Kim D. J., Davis C. L. (2015). Tumor endothelial cells with distinct patterns of TGFbeta-driven endothelial-to-mesenchymal transition. *Cancer Research*.

[11] Ranchoux B., Antigny F., Rucker-Martin C. (2015). Endothelial-to-mesenchymal transition in pulmonary hypertension. *Circulation*.

[12] Chen P. Y., Qin L., Baeyens N. (2015). Endothelial-to-mesenchymal transition drives atherosclerosis progression. *The Journal of Clinical Investigation*.

[13] Maddaluno L., Rudini N., Cuttano R. (2013). EndMT contributes to the onset and progression of cerebral cavernous malformations. *Nature*.

[14] Xu X., Tan X., Tampe B., Sanchez E., Zeisberg M., Zeisberg E. M. (2015). Snail is a direct target of hypoxia-inducible factor 1*α* (HIF1*α*) in hypoxia-induced endothelial to mesenchymal transition of human coronary endothelial cells. *The Journal of Biological Chemistry*.

[15] Chang C., Liu H., Wei C. (2017). Tongxinluo regulates expression of tight junction proteins and alleviates endothelial cell monolayer hyperpermeability via ERK-1/2 signaling pathway in oxidized low-density lipoprotein-induced human umbilical vein endothelial cells. *Evidence-Based Complementary and Alternative Medicine*.

[16] Chen Y., Li M., Zhang Y. (2018). Traditional Chinese medication Tongxinluo attenuates apoptosis in ox-LDL-stimulated macrophages by enhancing Beclin-1-induced autophagy. *Biochemical and Biophysical Research Communications*.

[17] Bai W. W., Xing Y. F., Wang B., Lu X. T., Wang Y. B., Sun Y. Y. (2013). Tongxinluo improves cardiac function and ameliorates ventricular remodeling in mice model of myocardial infarction through enhancing angiogenesis. *Evidence-Based Complementary and Alternative Medicine*.

[18] Cui H., Li X., Li N. (2014). Induction of autophagy by Tongxinluo through the MEK/ERK pathway protects human cardiac microvascular endothelial cells from hypoxia/reoxygenation injury. *Journal of Cardiovascular Pharmacology*.

[19] Wang J., Zhou J., Wang Y. (2017). Qiliqiangxin protects against anoxic injury in cardiac microvascular endothelial cells via NRG-1/ErbB-PI3K/Akt/mTOR pathway. *Journal of Cellular and Molecular Medicine*.

[20] Olayioye M. A., Neve R. M., Lane H. A., Hynes N. E. (2000). The ErbB signaling network: receptor heterodimerization in development and cancer. *EMBO Journal*.

[21] Doggen K., Ray L., Mathieu M., Mc Entee K., Lemmens K., De Keulenaer G. W. (2009). Ventricular ErbB2/ErbB4 activation and downstream signaling in pacing-induced heart failure. *Journal of Molecular and Cellular Cardiology*.

[22] Lemmens K., Doggen K., De Keulenaer G. W. (2007). Role of neuregulin-1/ErbB signaling in cardiovascular physiology and disease: Implications for therapy of heart failure. *Circulation*.

[23] Yarden Y., Sliwkowski M. X. (2001). Untangling the ErbB signalling network. *Nature Reviews Molecular Cell Biology*.

[24] Liu X., Gu X., Li Z. (2006). Neuregulin-1/erbB-Activation improves cardiac function and survival in models of ischemic, dilated, and viral cardiomyopathy. *Journal of the American College of Cardiology*.

[25] Xu M., Wu X., Jie B. (2014). Neuregulin-1 protects myocardial cells against H2 O2 -induced apoptosis by regulating endoplasmic reticulum stress. *Cell Biochemistry & Function*.

[26] Zhang J. F., Wei C., Wang H. T., Tang S. W., Jia Z. H., Wang L. (2013). Protective effect of qiliqiangxin capsule on energy metabolism and myocardial mitochondria in pressure overload heart failure rats. *Evidence-Based Complementary and Alternative Medicine*.

[27] Dixon J. A., Spinale F. G. (2011). Myocardial remodeling: Cellular and extracellular events and targets. *Annual Review of Physiology*.

[28] Vandekerckhove L., Vermeulen Z., Liu Z. Z. (2016). Neuregulin-1 attenuates development of nephropathy in a type 1 diabetes mouse model with high cardiovascular risk. *American Journal of Physiology-Endocrinology and Metabolism*.

[29] Li H., Zhao Q., Chang L. (2019). LncRNA MALAT1 modulates ox-LDL induced EndMT through the Wnt/*β*-catenin signaling pathway. *Lipids in Health and Disease*.

[30] Qin W., Du N., Zhang L. (2015). Genistein alleviates pressure overload-induced cardiac dysfunction and interstitial fibrosis in mice. *British Journal of Pharmacology*.

[31] Li X., Du N., Zhang Q. (2014). MicroRNA-30d regulates cardiomyocyte pyroptosis by directly targeting foxo3a in diabetic cardiomyopathy. *Cell Death & Disease*.

[32] Evrard S. M., Lecce L., Michelis K. C. (2016). Endothelial to mesenchymal transition is common in atherosclerotic lesions and is associated with plaque instability. *Nature Communications*.

[33] Lee S.-W., Won J.-Y., Kim W. J. (2013). Snail as a potential target molecule in cardiac fibrosis: paracrine action of endothelial cells on fibroblasts through snail and CTGF axis. *Molecular Therapy*.

[34] Schwartz B. G., Kloner R. A. (2012). Coronary no reflow. *Journal of Molecular and Cellular Cardiology*.

[35] Ma Q., Zhang S., Ning Y., Pu X., Yu G., Zheng Z. (2009). Effect of Tongxinluo on endothelial function and hypersensitive C-reactive protein in acute coronary syndrome patients undergoing percutaneous coronary intervention. *Zhong Nan Da Xue Xue Bao Yi Xue Ban*.

[36] Liu Y., Zou J., Li B. (2017). RUNX3 modulates hypoxia-induced endothelial-to-mesenchymal transition of human cardiac microvascular endothelial cells. *International Journal of Molecular Medicine*.

[37] Zou J., Liu Y., Li B. (2017). Autophagy attenuates endothelial-to-mesenchymal transition by promoting Snail degradation in human cardiac microvascular endothelial cells. *Bioscience Reports*.

[38] Shakeri H., Gevaert A. B., Schrijvers D. M. (2018). Neuregulin-1 attenuates stress-induced vascular senescence. *Cardiovascular Research*.

[39] Li J., Zhang Q., Ren C. (2018). Low-intensity pulsed ultrasound prevents the oxidative stress induced endothelial-mesenchymal transition in human aortic endothelial cells. *Cellular Physiology and Biochemistry*.

[40] Medici D., Potenta S., Kalluri R. (2011). Transforming growth factor-*β*2 promotes Snail-mediated endothelial—mesenchymal transition through convergence of Smad-dependent and Smad-independent signalling. *Biochemical Journal*.

[41] Zhang Y., Wu X., Li Y. (2016). Endothelial to mesenchymal transition contributes to arsenic-trioxide-induced cardiac fibrosis. *Scientific Reports*.

